# Outbreaks of Vector-Borne and Zoonotic Diseases Are Associated With Changes in Forest Cover and Oil Palm Expansion at Global Scale

**DOI:** 10.3389/fvets.2021.661063

**Published:** 2021-03-24

**Authors:** Serge Morand, Claire Lajaunie

**Affiliations:** ^1^CNRS ISEM—CIRAD ASTRE, Montpellier University, Montpellier, France; ^2^Faculty of Veterinary Technology, Kasetsart University, Bangkok, Thailand; ^3^Faculty of Tropical Medicine, Mahidol University, Bangkok, Thailand; ^4^Inserm-Laboratoire Population Environnement Développement (Aix-Marseille Université, IRD), Marseille, France

**Keywords:** infectious diseases, zoonoses, vector-borne diseases, deforestation, oil palm, public health, governance

## Abstract

Deforestation is a major cause of biodiversity loss with a negative impact on human health. This study explores at global scale whether the loss and gain of forest cover and the rise of oil palm plantations can promote outbreaks of vector-borne and zoonotic diseases. Taking into account the human population growth, we find that the increases in outbreaks of zoonotic and vector-borne diseases from 1990 to 2016 are linked with deforestation, mostly in tropical countries, and with reforestation, mostly in temperate countries. We also find that outbreaks of vector-borne diseases are associated with the increase in areas of palm oil plantations. Our study gives new support for a link between global deforestation and outbreaks of zoonotic and vector-borne diseases as well as evidences that reforestation and plantations may also contribute to epidemics of infectious diseases. The results are discussed in light of the importance of forests for biodiversity, livelihoods and human health and the need to urgently build an international governance framework to ensure the preservation of forests and the ecosystem services they provide, including the regulation of diseases. We develop recommendations to scientists, public health officers and policymakers who should reconcile the need to preserve biodiversity while taking into account the health risks posed by lack or mismanagement of forests.

## Introduction

The COVID-19 pandemic has called to investigate the consequences of biodiversity loss for the emergence of zoonotic diseases (1–4). Deforestation is a major cause of biodiversity loss (5) and the latest report on forests by United Nations Food and Agriculture Organization (FAO) and United Nations Environment Programme (UNEP) has emphasized the negative impact of deforestation on human health (6). The Sustainable Development Goals (SDG) of the United Nations (UN) specifically refers to the importance of forests. More precisely, the SDG 15 has two indicators with the first one that measures the proportion of the global forest area and the second one that assesses progress toward Sustainable Forest Management. The Aichi target 5 of the Convention on Biological Diversity (CBD) calls for a sharp decrease of the rate of loss of forests which should be to zero by 2020 (7). The Aichi targets 14 and 15, respectively, highlight the role of ecosystems in contributing to essential services and contributing to health as well as biodiversity conservation and climate change mitigation (7).

According to Curtis et al. (8) a quarter of global forest loss is due to the conversion of forest to produce commodities (beef, soy, palm oil, and wood fiber). The overall rate of this commodity-driven deforestation has not declined since 2001 (9). Forest conversion to commodities such as oil palm may affect several ecosystem functions such as carbon sequestration and soil regeneration (10). However, the loss of disease regulation during forest conversion has not been well-investigated. In Southeast Asia, a recent meta-analysis showed that increasing prevalence of vector-borne diseases such as dengue or chikungunya was associated with land conversion, including forests, to commercial plantations such as teak, rubber and oil palm (11). Nonetheless, it remains difficult to disentangle the respective influences of forest loss and conversion, other land use changes, demography, increased human and agricultural encroachments or the pressures of hunting on the rise of infectious diseases. Several studies have as exemplified that multiple factors are responsible of the outbreaks of Ebola in Africa (12, 13), Nipah (14) or *Plasmodium knowlesi* in Southeast Asia (15). Not only the emergence of new diseases, but also epidemics of infectious diseases appear to be linked to deforestation as recently evidenced for malaria epidemics in Brazil (16). Afforestation can also create new risks of infectious diseases, especially when it comes to commercial plantations. Abandon of agriculture land creates patchy matrix of shrubs that may also lead to new risk of zoonoses such as Lyme disease in the Northern America and Europe (17) or scrub typhus in Taiwan (18).

The present study is a first attempt to investigate at global scale whether the loss and gain of forest cover can promote outbreaks of vector-borne and zoonotic diseases. Here, we examine the global trends between changes in forest cover in recent decades and epidemics of human infectious diseases, using the GIDEON global database, which is the best available dataset on infectious diseases that has already been used in several studies (2, 19–21). We also examine the global pattern between increasing areas of oil palm and epidemics of human infectious diseases. We hypothesize that zoonotic and vector-borne diseases should be favored both by deforestation and by the increase of commodity plantations like oil palm, taking into account the concomitant increase in the size of the human population.

The results of our global study linking geography and infectious diseases are discussed in light of the importance of forests for biodiversity, livelihoods and human health and the need to urgently tackle the building of international governance to ensure the preservation of forests and the ecosystem services they provide, including the regulation of diseases.

## Materials and Methods

Data on forest cover (in share of total land area) by country from 1990 to 2016 were extracted from the World Bank (https://www.worldbank.org/). Data on oil palm harvested area by country were extracted from the FAOSTAT (http://www.fao.org/faostat/) of the Food and Agricultural Organization (FAO) of the United Nations. Data on population demography by country were also extracted from the World Bank (https://www.worldbank.org/).

Data on human infectious diseases were obtained from GIDEON (www.gideononline.com), which contains information on the occurrence of epidemics of human infectious diseases in each nation [see (2)]. In the GIDEON database, an outbreak follows the general definition of the WHO as any grouping of cases, including family clusters and epidemics. We used the classification of vector-borne and zoonotic diseases provided by Smith et al. (20). The 1990–2016 extracted dataset contains 3,884 outbreaks of 116 zoonotic diseases and 1,996 outbreaks of 69 vector-borne infectious diseases.

General additive modeling (GAM), an extension of the generalized linear models, was used to investigate the relationships between outbreaks of infectious diseases and forest cover or oil palm areas taking into account both the spatial autocorrelation and the temporal autocorrelation. The model assumes that the response variable is dependent on the univariate smooth-terms of independent variables (22). All models were fitted using the “MGCV” package (23). We used the function gam.check to choose the basis dimension for each predictor according to estimated degrees of freedom value in the main effect. Outputs of GAM models were obtained using the packages “gratia” (24) and “mgcViz” (25).

A first GAM was developed to investigate the number of outbreaks of zoonotic or vector-borne diseases as a function of forest cover, human population size, spatial distribution (using the country centroids) and time (year) using a negative binomial link function. This first model was:

g(E(outbreaks of infectious diseases)) = f1(long, lat) + f2(forest cover, year) + f3(human population size, year) + b

A second GAM was developed to investigate the number of outbreaks of zoonotic or vector-borne diseases as a function of oil palm areas, human population size, spatial distribution (using the country centroids) and time (year) using a negative binomial link function. This second model was:

g(E(outbreaks of infectious diseases)) = f1(long, lat) + f2(oil palm area, year) + f3(human population size, year) + b

Data manipulation and their representation were performed using “dplyr” and “ggplot2” (26) in R (27). Smooth regression was used to visualize the trend of change over time (28). We featured the correlations between the number of outbreaks, zoonotic and vector-borne diseases, and the changes in forest cover or oil palm areas, by country and over years, using the package “Hmisc” (29) in R. Maps were drawn using the package “rworldmap” (30).

## Results

**Figure 1:**
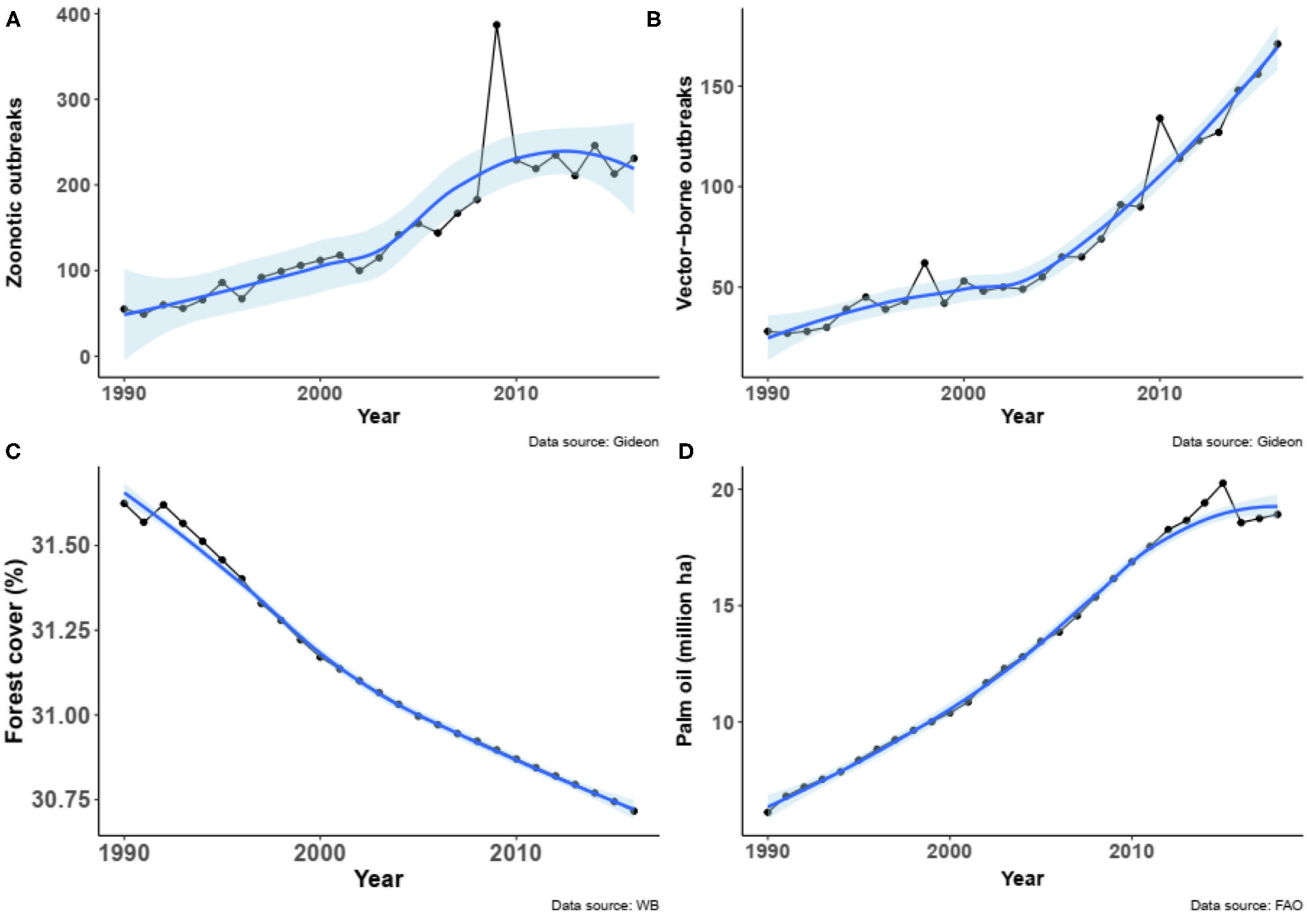
**(A)** Number of outbreaks of zoonotic diseases worldwide from 1990 to 2016 (data obtained from GIDEON). (**B)** Number of outbreaks of vector-borne diseases worldwide from 1990 to 2016 (data obtained from GIDEON). **(C)** Global change in forest cover (in share of global land) from 1990 to 2016 (data obtained from World Bank). **(D)** Relationship between the number of outbreaks of zoonotic diseases worldwide and the change in forest cover from 1990 to 2016. **(E)** Relationship between the number of outbreaks of vector-borne diseases worldwide and the change in forest cover from 1990 to 2016. Fitted smooth regressions (in blue) with confidence intervals (in light blue) are shown.

Because of the time limitation of the datasets, analyses were narrowed to the period from 1990 to 2016 for disease outbreaks (GIDEON), share of forest cover and area of oil palm (FAOSTAT) by country. Globally, the number outbreaks of zoonotic diseases increased from the period 1990–2016 (Spearman Rho = 0.99, *P* < 0.00001, *n* = 27, Figure 1A), as well as the number of outbreaks of vector-borne diseases (Spearman Rho = 0.94, *P* < 0.00001, *n* = 27, Figure 1B). In the same period of time, the forest cover slightly but significantly decreased worldwide (Spearman Rho = 0.91, *P* < 0.00001, *n* = 27, Figure 1C), while the total area of oil palm dramatically increased (Spearman Rho = 0.98, *P* < 0.00001, *n* = 27, Figure 1D).

**Figure 2:**
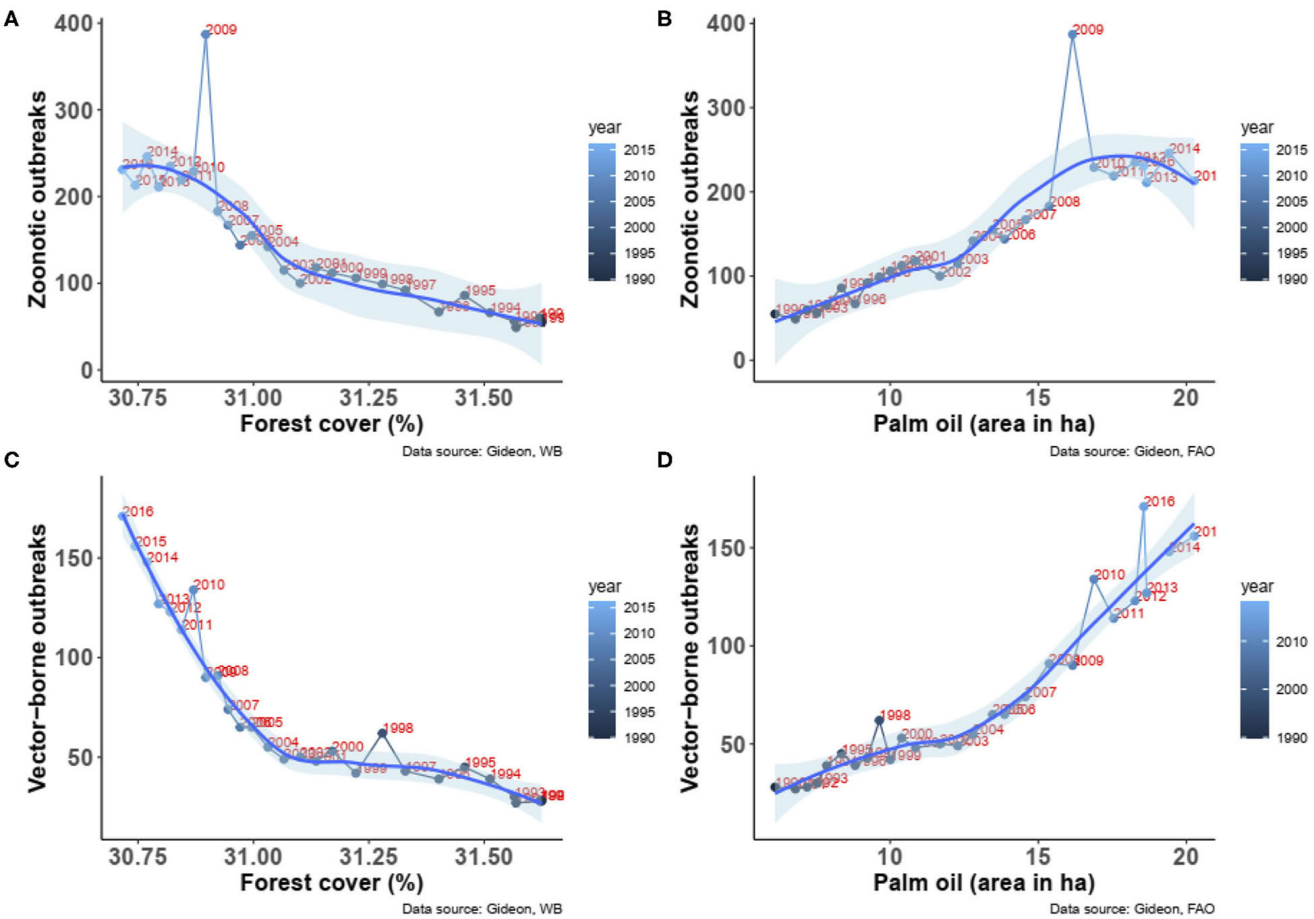
**(A)** Association between the change in forest cover and the number of outbreaks of zoonotic diseases from 1990 to 2016. **(B)** Association between the change in forest cover and the number of outbreaks of vector-borne diseases from 1990 to 2016. **(C)** Association between the change in palm oil areas and the number of outbreaks of zoonotic diseases from 1990 to 2016. **(D)** Association between the change in palm oil areas and the number of outbreaks of vector-borne diseases from 1990 to 2016. Fitted smooth regressions (in blue) with confidence intervals (in light blue) are shown (data from GIDEON and FAOSTAT).

The above global trends resulted in significant increases in both zoonotic diseases outbreaks (Spearman Rho = 0.92, *P* < 0.00001, *n* = 27, Figure 2A) and vector-borne diseases outbreaks (Spearman Rho = 0.94, *P* < 0.00001, *n* = 27, Figure 2B) in relation with the decrease in forest cover. Similarly, significant increases in both zoonotic (Spearman Rho = 0.91, *P* < 0.00001, *n* = 27, Figure 2C) and vector-borne diseases (Spearman Rho = 0.93, *P* < 0.00001, *n* = 27, Figure 2D) with the increase of oil palm area were observed.

**Figure 3:**
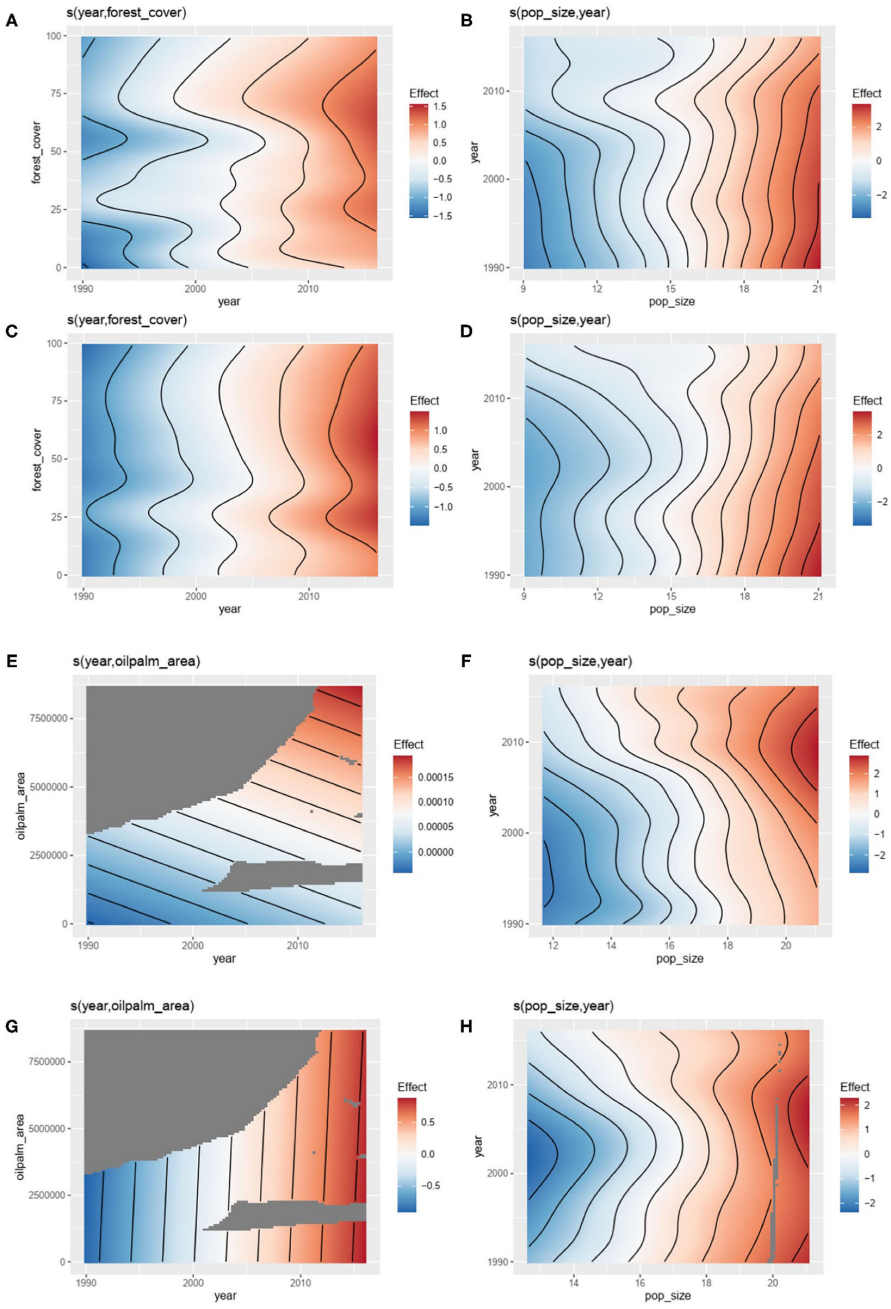
Results of General additive modeling (GAM) explaining: the number the number of outbreaks of zoonotic diseases with **(A)** forest cover (controlling for year) and **(B)** human population size (controlling for year) (see Table 1, model 1); the number the number of outbreaks of vector-borne diseases with **(C)** forest cover (controlling for year) and **(D)** human population size (controlling for year) (see Table 1, model 2); the number the number of outbreaks of zoonotic diseases with **(E)** oil palm area (controlling for year) and **(F)** human population size (controlling for year) (see Table 1, model 3); the number the number of outbreaks of vector-borne diseases with **(G)** oil palm area (controlling for year) and **(H)** human population size (controlling for year) (see Table 1, model 4) (data from GIDEON, FAOSTAT and World Bank).

Relationships by country were investigated using GAM. The first GAM investigating the number of outbreaks of zoonotic or vector-borne diseases in humans explained respectively 53.4% [*R*^2^ (adj) = 0.65] and 40.5%; [*R*^2^ (adj) = 0.49] of the deviance (Table 1) with a significant influence of the forest cover (*P* < 0.00001, Table 1, Figures 3A,C) and human population size (*P* < 0.00001, Table 1, Figures 3B,D) taking into account the effect of year and the spatial autocorrelation (*P* < 0.00001, Table 1).

**Table 1:** Results of general additive modeling (GAM) explaining the number of outbreaks of infectious diseases, zoonotic or vector-borne, per country and per year, with forest cover or oil palm areas, human population size and the matrix of longitude/latitude of country centroids as independent variables, using a negative binomial link and controlling for spatial and temporal effects, with approximate significance of smooth terms (edf, estimated residual degrees of freedom; df, degree of freedom) (see Methods for the models, data from GIDEON, FAOSTAT and World Bank) (see Figure 3).

**Response variable**	**Explanatory variables**	**edf**	**Chi^**2**^ (df)**	***P*-value**	**Deviance (in%), *R*^**2**^ adjusted, n**
1. Outbreaks of zoonotic diseases	(Forest cover, year)	19.7	106 (29)	<0.0001	
	(Population size, year)	19.9	1,250 (28)	<0.0001	
	(Longitude, latitude)	24.1	645 (29)	<0.0001	53.4, 0.65, 5311
2 Outbreaks of vector-borne diseases	(Forest cover, year)	13.4	52.5 (29)	<0.0001	
	(population size, year)	17.0	619.8 (28)	<0.0001	
	(Longitude, latitude)	21.2	348.1 (29)	<0.0001	40.5, 0.49, 4784
3. Outbreaks of zoonotic diseases	(Oil palm area, year)	0.001	0.0 (29)	0.54	
	(Population size, year)	15.1	488 (28)	<0.0001	
	(Longitude, latitude)	11.7	161 (29)	<0.0001	55.1, 0.65, 1126
4. Outbreaks of vector-borne diseases	(Oil palm area, year)	1.74	6.79 (29)	0.0029	
	(Population size, year)	11.05	127.34 (28)	<0.0001	
	(Longitude, latitude)	11.25	81.74 (29)	<0.0001	49.3; 0.54, 1072

The second GAM model investigating the number of outbreaks of zoonotic or vector-borne diseases in humans explained, respectively, 55.1% [*R*^2^ (adj) = 0.65] and 49.3% [*R*^2^ (adj) = 0.54] of the deviance (Table 1) with a significant influence of human population size (*P* < 0.00001, Table 1, Figures 3G,H) taking into account the effect of year and the spatial autocorrelation (*P* < 0.00001, Table 1). However, the influence of the oil palm area was only significant for vector-borne diseases' outbreaks (*P* < 0.00001, Table 1, Figure 3F) and not for zoonotic diseases' outbreaks (*P* = 0.54, Figure 3E).

**Figure 4:**
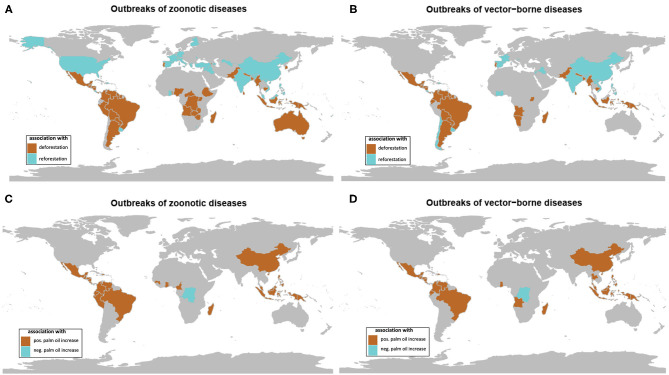
Maps of significant association between forest cover change (deforestation in brown, reforestation in blue) and **(A)** outbreaks of zoonotic diseases and **(B)** outbreaks of vector-borne diseases on temporal trends from 1990 to 2016. Maps of significant association between oil palm area change and **(C)** outbreaks of zoonotic diseases and **(D)** outbreaks of vector-borne diseases (positive association in brown, negative association in blue) on temporal trends from 1990 to 2016 (data from GIDEON and FAOSTAT) (see Supplementary File).

The investigation by country showed negative and positive associations (using Spearman correlation test) between the number of outbreaks of both zoonotic and vector-borne diseases and the changes in forest cover from 1990 to 2016 (Figures 4A,B). Significant associations between forest cover loss and zoonotic and vector-borne disease outbreaks were observed, respectively, for 47 and 49 countries, with the majority in tropical climate (Annex 1, Supplementary Table 1). Significant associations between forest cover gain and zoonotic or vector-borne outbreaks were observed for, respectively, 27 and 29 countries, the majority situated outside the tropical environment (see Annex 1, Supplementary Table 2).

**Figure 5:**
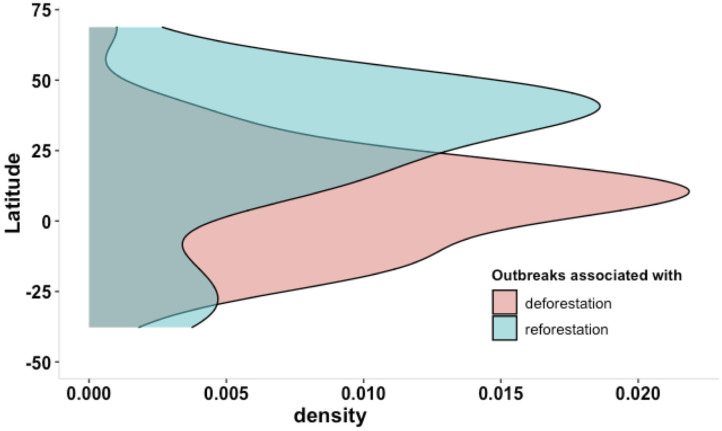
Density plot of significant association between outbreaks of zoonotic and vector-borne diseases with deforestation (in red) or reforestation (in green) in relation to latitudinal gradient.

Taking the example of vector-borne diseases, two groups of countries can be characterized. The first group of countries situated in tropical regions showed relatively high forest cover and high forest loss in association with increasing outbreaks (Figure 5). The second group of countries situated mostly in temperate zone showed relatively low forest cover and forest gain in association with increasing outbreaks (Figure 5).

## Discussion

The results of the present analysis are in agreement with previous claims that linked emergence of zoonotic and vector-borne diseases with deforestation (31, 32). Both zoonotic and vector-borne diseases showed an increase in the number of their outbreaks that appear to be linked with deforestation globally over the period of 1990 to 2016, with vector-borne diseases showing a most dramatic increase over the last years of the period considered. Our results also show a positive association between the number of vector-borne disease outbreaks and the increase in land areas converted to oil palm plantations. Reforestation may be also of concern by an increasing number of recorded epidemics, especially in non-tropical countries with low or moderate forest cover but also in countries with strong reforestation policies such as China and Vietnam.

### Deforestation and Disease Outbreaks

The significant associations observed between epidemics and deforestation mostly concerned the countries of the intertropical zone with high forest cover, such as Brazil, Peru, and Bolivia in South America, Democratic Republic of Congo and Cameroon in Africa, Indonesia, Myanmar and Malaysia in Southeast Asia, among others.

Several studies have already attested that deforestation has caused malaria epidemics in South America and that forest clearing had favored the mosquito vector *Anopheles darlingi* in Southeast Asia for the species complex *A. dirus, A. minimus, A. balabacensis* (33). Association between deforestation and outbreaks of malaria has been documented in Brazil (16). A comparative analysis of vector and non-vector species showed that the net effect of deforestation favors mosquitoes that serve as vectors of human diseases (34). Re-emergence of arthropod-borne leishmaniasis has also been put in relation with deforestation (35). The ecological mechanism proposed is that forest fragmentation, forest conversion and biodiversity loss lead to the loss of ecological regulation of small mammals, which are main reservoirs of *Leishmania* species (36). Moreover, zoophilic sandflies vectors have shown their ability to adapt to human blood and to human dwellings (peridomestic or synthropic behavior) (33). Altogether deforestation and biodiversity regulation loss favor reservoir and/or vector populations, affect disease transmission dynamics and ultimately lead to increasing human contacts with vectors or reservoirs. Several studies have emphasized the role of forest deforestation in the emergence of zoonotic diseases such as Ebola in Africa (12, 13).

Our results find support from the recent study of Gibb et al. (37), which demonstrates how the global land use changes, including forest conversion, may favor zoonotic reservoirs and the risks of zoonotic diseases.

### Reforestation and Epidemics

Reforestation can increase biodiversity loss when forest expansion is made at the expense of grasslands, savannas, and open-canopy woodlands (38, 39). Afforestation and forest expansion can then decrease ecosystem services (39). According to the United Nations Food and Agriculture Organization (FAO) forests are land with a tree-canopy cover of more than 10%, which does not help to distinguish old-growth grasslands from anthropogenic vegetation neither natural forest expansion from forest regeneration nor plantations (39–41).

Our results clearly suggest that it is not only forest clearance that is responsible of outbreaks of infectious diseases (42), but also reforestation or afforestation, particularly in countries outside the tropical zone. However, the data used does not differentiate between the different kinds of reforestation: plantation for commodities, afforestation, or abandonment of agricultural lands.

Temperate and tropical countries with grassy biomes at risk of afforestation and forest expansion according to Veldman et al. (39) such as USA, European countries, China, Turkey, India or Vietnam are the ones showing a positive association between reforestation and disease transmission. Hence, the increase in incidence of tick-borne encephalitis in humans in Italy was explained by the ratio of coppice to high stand forest in Italy with natural reforestation that may favor the abundance of the small mammal reservoirs of tick-borne viruses (43). Similarly, reforestation and rebounding deer populations created ecological conditions for tick vectors and the reemergence of tick-borne diseases in USA (44).

Investigation of the trends in forest and agricultural land covers in several Asian countries from 1962 to 2011 showed that China, India, Philippines and Vietnam had a net increase in forest cover, with a parallel increase in the area of agricultural land in China and India, while Indonesia, Laos, and Malaysia had a net decrease in forest cover, with a consistent decrease in agricultural land in Malaysia (45). The results of our analysis fit quite well with these forest cover trends. The recent study of Sloan et al. (46) confirmed that major planted area expansion occurs in Southeast Asia alongside minor net forest cover change.

### Oil Palm and Epidemics

Vijay et al. (47) have emphasized the negative impact of oil palm expansion on biodiversity, especially in Southeast Asia and South America in comparison to Africa. Our results clearly show an association between the increasing number of outbreaks of vector-borne diseases and the increase of oil palm plantations. Interestingly, among them there were countries that were not affected by deforestation, such as Thailand, or that actively promoted reforestation, such as China and Vietnam.

A meta-analysis quantified the exposure to infectious diseases in relation to land uses in Southeast Asia showing a strong effect for oil palm monoculture on the risks of infectious diseases, either zoonotic or vector-borne (46). Strong effects of oil palm monoculture were also observed for rickettsial diseases (scrub typhus, spotted fever group) and malaria. Studies have also documented increase of mosquito-borne viruses, such as *Aedes albopictus* and *Aedes aegypti*, in oil palm and rubber plantations (48, 49) favoring the spread of dengue, zika, chikungunya, and yellow fever. In the same manner, the populations of generalist vectors of *Trypanozoma cruzi*, the agent of Chagas disease, better developed in oil palm plantations in Columbia (50).

### Bias and Missing Factors

Several potential biases may limit the analyzes presented in this study. First, the registration of infectious diseases depends on several factors such as expenditure on public health services and the quality of the health surveillance system, which vary from country to country but also from year to year in relation to financial priorities, development aid, private sector participation, among others (2). However, our analysis was limited from years 1990 to 2016 with a noticed improvement in global health surveillance. Second, as already mentioned, the definition of a forest by the FAO (30) does not help to distinguish the different types of forests. Third, there are potential confounding effects of climate change especially on vector-borne diseases such as dengue (51) and other vector-borne diseases. For example, the increase of incidence of scrub typhus in China and Northern Thailand was found correlated with the increase of mean temperature (52). Several other factors are potential drivers of emergence or of epidemics of zoonotic and vector-borne diseases such as livestock (2, 53), pets (54–57) and trade (58). The fact that many factors play important role in the emergence and epidemics calls for the development of more integrative research (59–61).

### International Governance of Forests and Their Contributions to Healthy Planet and People

Institutionally, the increase in epidemics due to land change and deforestation is acknowledged in the joint report of the Secretariat of the CBD and the WHO (62). While forests have been on the international legal agenda since the 1960s, the Earth Summit of 1992 failed to produce a convention on forests due to the diverging interests between developed countries claiming that forests should be considered as a global common on the one hand, and developing countries asserting that forests are a sovereign national resource (63) on the other hand. Nevertheless, the Earth Summit produced a declaration on “forest principles” (Non-legally Binding Authoritative Statement of Principles for a Global Consensus on the Management, Conservation and Sustainable Development of All Types of Forests) which gave birth to the Intergovernmental Forum on Forests (Intergovernmental Panel on Forest in 1992) notably to implement the forest principles and the Chapter 11 of the Agenda 21 on Deforestation in 1992 (64). Apart from the 1983 international agreement, renewed in 1994 and 2006, on tropical timber harvesting, the International Tropical Timber Agreement organizing the work of the International Tropical Timber Organization, no other international convention has been devoted exclusively to forests. This text does not mention biodiversity but focuses on the promotion of the sustainable management of tropical timber and the expansion and diversification of International trade in tropical timber (Article 1, ITTO 2006) (65).

In 2011, the Bonn Challenge made the goal to restore 150 million hectares of forest by 2020. It has been coupled with a joint initiative between ITTO and the CBD to enhance biodiversity conservation in tropical forests with the direct participation of local stakeholders, addressing the main drivers of biodiversity loss in tropical forests: deforestation and forest degradation (ITTO CBD, 2011). In 2014, The New York Declaration on Forests (66) endorsed the Bonn Challenge goal. The Declaration on Forests commits to halving the rate of deforestation by 2020, ending it by 2030, and restoring hundreds of millions of hectares of degraded land. However, the Declaration on Forests is based on voluntary, non-legally binding political declaration between governments, businesses and representatives of civil society [on the authoritative but non-legally binding principles (67)]. The World Commission on Forests and Sustainable Development created in 1995 authored a report in 2000 “Our forests, our future” which advocated for the use of the Forest Capital Index using different indicators of forest qualities such as: surface area; standing biomass; net primary productivity; species richness and diversity; age class of trees; leaf area index; soil fertility measures; soil organic matter content; and the health of forest stands. Forest health is defined as a characteristic of forests that are naturally resilient to damage based on factors such as biological diversity and ecosystem stability (68). Recently, Dobson et al. (1) have called for major investments to prevent tropical deforestation as a way to protect against future zoonosis outbreaks, but incidentally without referring to any international declarations and engagements and particularly the Bonn Challenge. However, the Second Bonn Challenge progress report (69) mentioned only two times the word “health” and only in reference to healthy environment and the word “disease” was used only for forest disease without mentioning human disease.

### Recommendations for Science and Policy

Based on the results of our study, two main outputs should be stated. First, emerging zoonotic and vector-borne diseases are important threats, but it is also crucial to acknowledge the high burdens of neglected zoonotic and vector borne diseases in tropical countries. Second, while our study gives new support for a link between global deforestation and outbreaks of zoonotic and vector-borne diseases, our study also evidences that reforestation and plantations may also contribute to epidemics of infectious diseases.

In 2015, representatives of civil society from across Asia, Africa, and Latin America called on the European Parliament to halt the demand for biofuels in Europe and refrain from using biofuels derived from oil palm plantations considering that industrial oil palm plantations are one of the world's largest contributors to greenhouse gas emissions. While the European Union proposes to phase out palm oil-based biofuels by 2030, Malaysia follows Indonesia and prepares dispute settlement proceedings before the World Trade Organization against the European Union's proposal which constitutes a damage to the oil palm industry. Our result shows that oil palm plantations may also constitute a threat to global health by favoring zoonotic and vector-borne diseases.

Then, what is needed is a better way to stop both the loss of biodiverse native forests and a better management of afforestation to increase their contribution not only to biodiversity, or carbon sequestration, but to local livelihood and health.

Scientists, public health and policymakers should reconcile the need to preserve biodiversity while taking into account the health risks posed by lack or mismanagement of forest (both deforestation and afforestation) by considering the following recommendations:

- halt deforestation by implementing an international governance of forests and their contributions to healthy planet and people;
- develop research on disease regulating service provided by forests and other ecosystems, which may help at better manage forested and planted areas;
- recognize that forests and plantations not only contribute to carbon sequestration but to biodiversity and global health;
- following Veldman et al. (39), revise the forest definitions of the FAO as to avoid afforestation, forest expansion, and agricultural conversion of grasslands.

Finally, and taking the words of Humphreys (70), a democratic and publicly accountable global governance is needed to “address the deep causes of deforestation, principally predatory and unregulated corporations that profit from the degradation and destruction of forest public goods.”

## Data Availability Statement

All data are available from GIDEON, FAOSTATS and World Bank. Raw statistics for Figure 4 were given in Supplementary File.

## Author Contributions

SM gathered the data and performed the analyses. SM and CL wrote the manuscript. All authors contributed to the article and approved the submitted version.

## Conflict of Interest

The authors declare that the research was conducted in the absence of any commercial or financial relationships that could be construed as a potential conflict of interest.
